# Метастатическое поражение надпочечников. Опыт НМИЦ эндокринологии

**DOI:** 10.14341/probl13195

**Published:** 2023-08-30

**Authors:** Н. В. Пачуашвили, А. А. Рослякова, Э. Э. Порубаева, Д. Г. Бельцевич, А. Н. Гадзыра, Н. А. Дрогалов, А. А. Лазарева, Л. С. Урусова

**Affiliations:** Первый Московский государственный медицинский университет им. И.М. Сеченова (Сеченовский Университет); Национальный медицинский исследовательский центр эндокринологии; Национальный медицинский исследовательский центр эндокринологии; Национальный медицинский исследовательский центр эндокринологии; Национальный медицинский исследовательский центр эндокринологии; Национальный медицинский исследовательский центр эндокринологии; Национальный медицинский исследовательский центр эндокринологии; Национальный медицинский исследовательский центр эндокринологии

**Keywords:** метастазы, надпочечники, иммуногистохимическое исследование

## Abstract

Дифференциальная диагностика между доброкачественными, первичными и вторичными злокачественными опухолями является критической проблемой в клиническом лечении опухолей надпочечников, особенно у пациентов с изолированными поражениями надпочечников. В большинстве случаев правильный диагноз удается установить микроскопически при стандартной окраске гематоксилином и эозином. Однако бывают случаи, когда почти невозможно отличить метастаз от первичного рака надпочечников, поэтому для постановки диагноза требуется проведение иммуногистохимического исследования.В данной статье приводится пять уникальных наблюдений вторичных опухолей надпочечников, которые были диагностированы нами в текущем операционном материале: метастаз светлоклеточного почечно-клеточного рака, фолликулярного варианта папиллярного рака щитовидной железы, метастаз ороговевающей плоскоклеточной карциномы шейки матки, лимфоэпителиомоподобной карциномы мочевого пузыря, а также злокачественной мезотелиомы. Учитывая крайнюю редкость представленных наблюдений, приводим анализ данных литературы.

## Abstract (en)

Differentiation between benign, primary and secondary malignant tumors is a critical problem in the clinical treatment of adrenal tumors, especially in patients with isolated adrenal lesions. In most cases, the correct diagnosis can be established microscopically with standard staining with hematoxylin and eosin. However, there are cases when it is almost impossible to distinguish metastasis from primary adrenal cancer, so an accurate diagnosis requires an immunohistochemical examination.This article presents five unique observations of secondary adrenal tumors that were diagnosed by us in the current surgical material: metastasis of clear cell renal cell carcinoma, follicular variant of papillary thyroid cancer, metastasis of keratinizing squamous cell carcinoma of the cervix, lymphoepithelioma-like carcinoma of the bladder, as well as malignant mesothelioma. Taking into account the extreme rarity of the presented observations, we present an analysis of the literature data.

## АКТУАЛЬНОСТЬ

Опухоли надпочечников размером 1 см и более в диаметре обнаруживают приблизительно у 1% населения. Большинство инциденталом надпочечников являются доброкачественными функционально неактивными аденомами, однако могут обнаруживаться и злокачественные новообразования [1].

Значительный рост заболеваемости и трудности дифференциальной диагностики опухолей надпочечников обуславливают повышенный интерес к данной патологии. Надпочечники являются распространенным местом метастазирования некоторых злокачественных новообразований из-за богатого синусоидального кровоснабжения [2]. Согласно данным серий аутопсий установлено, что вторичное поражение надпочечника при наличии онкологического анамнеза обнаруживается в 32–73% случаев [3].

Из-за значительных различий в лечебной тактике принципиально важными являются диагностика и дифференцирование метастатического процесса от первичных опухолей надпочечников.

Цель данного исследования — в клинико-морфологическом анализе уникальных случаев обнаружения метастатического поражения надпочечников, которые были диагностированы в Национальном медицинском исследовательском центре эндокринологии Минздрава России за 7-летний период наблюдения с 2015 по 2022 гг.

## КЛИНИЧЕСКИЙ СЛУЧАЙ №1

Пациентка И., 63 года. В анамнезе левосторонняя нефрэктомия по поводу светлоклеточного рака почки, рТ3аN0Mx, выполненная осенью 2020 г. Тогда же впервые было выявлено образование в проекции правого надпочечника размерами 42×26×41 мм, с высокой нативной плотностью.

По данным мультиспиральной компьютерной томографии (МСКТ) в марте 2022 г. выявлена отрицательная динамика в виде увеличения размеров опухоли правого надпочечника до 62×69×49 мм, с высокой нативной плотностью +30…+40 HU, плотностью по фазам сканирования 100–105–60 HU, коэффициент относительного вымывания — 42,8%, абсолютного — 69%. По результатам лабораторного обследования данных в пользу гормональной активности не получено: 1) в суточной моче, собранной с консервантом, метанефрин — 218,4 мкг/сут (25–312), норметанефрин — 443,4 мкг/сут (35–445); 2) альдостерон — 102,51 пмоль/л (70,9–980), ренин — 10,12 МЕ/л (2,8–39,9), калий — 4,77 ммоль/л; 3) АКТГ базальный — 1,11 пмоль/л (1,6–13,9), кортизол в ходе ночного подавляющего теста с 1 мг дексаметазона — 12,0 нмоль/л (менее 50). По данным позитронно-эмиссионной томографии с компьютерной томографией (ПЭТ/КТ) всего тела с 18F-фтордезоксиглюкозой (18F-ФДГ) отмечается фоновая метаболическая активность образования правого надпочечника, вторичного поражения других органов и систем не выявлено. Заключение: Образование правого надпочечника (злокачественного КТ-фенотипа; адренокортикальный рак? метастаз? феохромоцитома?).

Учитывая высокий злокачественный потенциал образования в проекции правого надпочечника, выполнено хирургическое лечение в объеме правосторонней адреналэктомии с опухолью. При ревизии правый надпочечник представлен опухолевидным образованием размером до 6 см в диаметре сероватого цвета, обильно васкуляризованным.

По результатам патолого-анатомического исследования надпочечник с опухолью и жировой клетчаткой размерами 90×45×50 мм. На разрезе к надпочечнику прилежит опухоль дряблой консистенции пестрого вида с желтовато-сероватыми, белесыми и бурыми участками. В центре полупрозрачный желтоватый участок, по периферии имеется кистозная полость, опухоль размерами 5,5 см в наибольшем измерении, с четкими ровными контурами, окружена тонкой капсулой (рис. 1а). Гистологическое исследование: в надпочечнике неравномерной толщины с относительно сохранными слоями коркового вещества и мозговым веществом наблюдается рост опухоли из крупных полиморфных светлых клеток альвеолярного строения с очагами кистозной дегенерации, свежих и старых кровоизлияний (рис. 1б). Результаты иммуногистохимического (ИГХ) исследования: клетки опухоли не экспрессируют SF-1, Melan A, Inhibin A и Chromogranin A. Отмечается выраженная положительная реакция на PAX2, цитокератины (СК 8/18), RCC, CD10 и виментин. С учетом клинико-анамнестических данных опухолевый рост можно интерпретировать как метастаз светлоклеточного почечно-клеточного рака (ПКР) в надпочечник.

**Figure:**
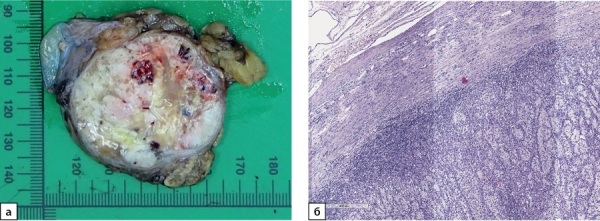
Рисунок 1а. Макропрепарат надпочечника с метастазом светлоклеточного почечно-клеточного рака диаметром 5,5 см.Рисунок 1б. Метастаз светлоклеточного почечно-клеточного рака в надпочечник. Опухоль представлена крупными полиморфными светлыми клеткамиальвеолярного строения с очагами кистозной дегенерации. Ув. ×100. Окраска гематоксилином и эозином.

## КЛИНИЧЕСКИЙ СЛУЧАЙ №2

Пациентка П., 66 лет. Из анамнеза известно, что более 20 лет назад была оперирована по поводу высокодифференцированного папиллярного рака щитовидной железы (ПРЩЖ) в объеме тиреоидэктомии.

В 2015 г. при проведении МСКТ органов грудной клетки по поводу пневмонии выявлено очаговое поражение нижней доли левого легкого, произведена атипичная резекция нижней доли левого легкого в связи с подозрением на метастатическое поражение. По результатам патологоанатомического исследования операционного материала верифицирован метастаз высокодифференцированного рака щитовидной железы в ткани легкого.

Получила 4 курса радиойодтерапии (последний в августе 2019 г., 6 ГБк). На посттерапевтической сцинтиграфии с технецием-99м-пертехнетатом с ОФЭКТ-КТ — верхнемедиастинальный лимфоузел максимальным размером до 1 см, требующий дифференцировки. Других признаков структурной прогрессии не выявлено.

По данным ПЭТ/КТ с 18F-ФДГ в режиме «все тело» в 2020 г. впервые определяется появление гиперваскулярного образования с гиперфиксацией радиофармпрепарата примерными размерами 31×21 мм, уровень максимального стандартизированного накопления радиофармпрепарата (SUVmax) — 5,73, в проекции правого надпочечника. Данных в пользу других гиперметаболических образований не получено. С учетом высоких цифр тиреоглобулина крови — 405,9 пг/мл и отсутствия других очагов, подозрительных в отношении вторичного поражения, заподозрен солитарный метастаз ПРЩЖ в правый надпочечник. По результатам обследования данных в пользу гормональной активности не получено: 1) в суточной моче, собранной с консервантом, метанефрин — 130,8 мкг/сут (25–312), норметанефрин — 342 мкг/сут (35–445); 2) альдостерон — 112,51 пмоль/л (70,9–980), ренин — 12,3 МЕ/л (2,8–39,9), калий — 4,77 ммоль/л; 3) АКТГ базальный — 7,4 пмоль/л (1,6–13,9), кортизол в ходе ночного подавляющего теста с 1 мг дексаметазона — 42,0 нмоль/л (менее 50).

Пациентке был выставлен основной диагноз: гормонально-неактивное образование правого надпочечника с высоким злокачественным потенциалом. В связи с этим проведена лапароскопическая правосторонняя адреналэктомия с опухолью. Интраоперационно выявлено, что надпочечник содержит опухоль темно-вишневого цвета диаметром 3,5 см.

По результатам патолого-анатомического исследования в постоперационном материале обнаружен метастаз фолликулярного варианта ПРЩЖ (рис. 2а, 2б). При ИГХ-исследовании клетки опухоли экспрессируют тиреоглобулин (рис. 2в) и TTF1 (рис. 2г), не экспрессируют напсин А. Индекс пролиферации Ki67 составляет около 7%. Послеоперационный уровень ТГ, определенный через 6 мес после оперативного вмешательства, составил 1,4 пг/мл.

**Figure:**
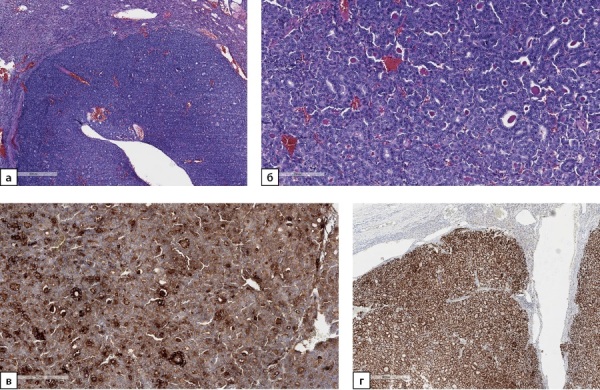
Рисунок 2а. Метастаз фолликулярного варианта папиллярного рака щитовидной железы в надпочечник. Ув. ×100. Окраска гематоксилином и эозином.Рисунок 2б. Метастаз фолликулярного варианта папиллярного рака щитовидной железы в надпочечник. Опухоль представлена фолликулами разного размера, выстланными клетками с большими неправильными тесно расположенными ядрами. Ув. ×200. Окраска гематоксилином и эозином.Рисунок 2в. Метастаз фолликулярного варианта папиллярного рака щитовидной железы в надпочечник. Экспрессия тиреоглобулина опухолевыми клетками. Ув. ×200. ИГХ-реакция с антителом к тиреоглобулину.Рисунок 2г. Метастаз фолликулярного варианта папиллярного рака щитовидной железы в надпочечник. Позитивное окрашивание опухолевых клеток в реакции с антителом к TTF1. Ув. ×100. ИГХ-реакция с антителом к TTF1.

## КЛИНИЧЕСКИЙ СЛУЧАЙ №3

Пациентка Г., 65 лет, с установленным диагнозом рака шейки матки, pТ2bNхМ0. Получила комбинированную терапию: 2015 г. — сочетанное лучевое лечение на фоне химиотерапии Фтора-фур, 2019 г. — торакоскопическая резекция верхней и нижней долей правого легкого по поводу mts, 2020 г. — стереотаксическая дистанционная лучевая терапия рецидива заболевания в области корня правого легкого.

При очередном динамическом обследовании по данным ПЭТ/КТ всего тела с 18F-ФДГ от 07.12.2021 выявлена картина объемного образования в проекции правого надпочечника размерами 38×22 мм, с высоким захватом радиофармпрепарата, иных очагов гиперметаболизма не выявлено. По результатам МСКТ от 10.01.2022 в проекции правого надпочечника определяется объемное образование размерами 45×24 мм, высокой нативной плотности +20…+45 HU. Отмечается неравномерное накопление контрастного препарата, преимущественно в периферических отделах. На протяжении 17 мм наружный контур образования интимно прилежит к правой доле печени, четкой границы между ними не отмечается. По результатам лабораторного обследования 12.2021–01.2022 альдостерон — 283 пг/мл (25,2–392), ренин — 16,1 мкМЕ/мл (4,4–46,1), кортизол (утро) — 399 нмоль/л (101–535), в суточной моче, собранной с консервантом, метанефрин общий — 71,68 мкг/сут (18–277), норметанефрин общий — 198,8 мкг/сут (42–423). Наличие артериальной гипертонии отрицает. Таким образом, у пациентки с отягощенным онкологическим анамнезом выявлено солитарное образование в проекции правого надпочечника с высоким злокачественным потенциалом.

Интраоперационно при ревизии правый надпочечник у верхнего полюса почки желтоватого цвета, в латеральной ножке надпочечника определяется опухолевидное образование до 4,5 см в диаметре. Выполнена лапароскопическая правосторонняя адреналэктомия с опухолью.

Результаты патолого-анатомического исследования: в ткани надпочечника определяется новообразование, представленное ороговевающим плоским эпителием с большим количеством митозов, в том числе и патологических, обширными очагами некроза и с наличием комедонекрозов, распространением за пределы надпочечника в прилежащую жировую клетчатку (рис. 3). При ИГХ-исследовании отмечается отсутствие экспрессии SF-1, Melan A, Inhibin A и Chromogranin A. Положительная реакция на CK5/6, p63. С учетом клинико-анамнестических данных и морфологической картины опухоль является метастазом ороговевающей плоскоклеточной карциномы шейки матки в надпочечник.

**Figure:**
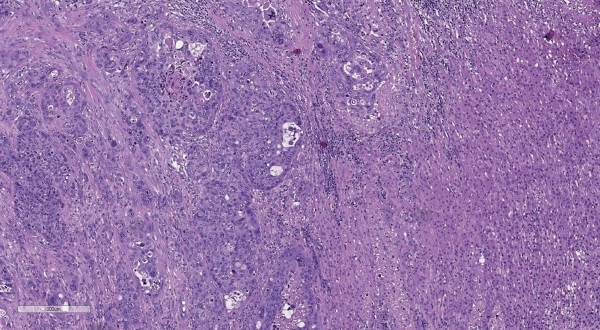
Рисунок 3. Метастаз ороговевающей плоскоклеточной карциномы шейки матки в надпочечник. Опухоль представлена ороговевающим плоским эпителием с большим количеством патологических митозов и обширными очагами некроза. Ув. ×100. Окраска гематоксилином и эозином.

## КЛИНИЧЕСКИЙ СЛУЧАЙ №4

Пациент Т., 65 лет. Из анамнеза известно, что в 2011 г. оперирован в объеме трансуретральной резекции по поводу опухоли мочевого пузыря; по результатам патолого-анатомического исследования операционного материала верифицирован папиллярный переходноклеточный рак низкой степени злокачественности, без инвазии в подслизистый слой, pT1aN0M0. Принимая во внимание наличие онкологического заболевания в анамнезе, с 2011 г. находится на диспансерном учете.

При очередном обследовании в 2015 г. по данным МСКТ выявлено объемное образование в проекции правого надпочечника с четкими, ровными контурами, размерами 17×25×27 мм, мозаичной нативной плотности +17…+31 HU. В ходе динамического наблюдения по данным контрольной МСКТ в 2017 г. отмечено увеличение размеров объемного образования в проекции правого надпочечника до 40,7×35×44,3 мм, с четкими, несколько неровными контурами, плотностью до 37 HU, неоднородной структуры за счет кальцинатов и гиподенсного участка плотностью до 17 HU. Образование неравномерно накапливает контрастный препарат до 70 HU в артериальную фазу, в венозную — 71 HU, в отсроченную — 68 HU (9%). Данных в пользу наличия в грудной клетке, брюшной полости и малом тазу других объемных образований с высоким злокачественным потенциалом не получено. По результатам лабораторного обследования данных в пользу гормональной активности не получено: 1) в суточной порции мочи, собранной с консервантом, метанефрин — 87,88 мкг/сут (25–312), норметанефрин — 175,5 мкг/сут (35–445); 2) альдостерон — 82,7 пмоль/л (70,9–980), ренин — 9,541 МЕ/л (2,8–39,9), калий — 4,2 ммоль/л; 3) АКТГ базальный — 13,09 пг/мл (7–66), кортизол свободный в суточной порции мочи — 262,756 нмоль/сут (60–413).

Учитывая высокий злокачественный потенциал образования в проекции правого надпочечника, выполнено хирургическое лечение в объеме правосторонней адреналэктомии с опухолью. В ткани атрофичного правого надпочечника выявлена опухоль темного цвета, диаметром 4,0 см с зонами некроза (рис. 4а).

По результатам патолого-анатомического исследования к надпочечнику обычного гистологического строения прилежит опухоль солидного строения, состоящая из крупных эпителиальных клеток с бледной цитоплазмой (рис. 4б), экспрессирующих multi-cytokeratin (MCK) (рис. 4в) и p53, строма опухоли состоит из нежноволокнистой соединительной ткани, инфильтрированной лимфоидными элементами, экспрессирующими CD45 (рис. 4г). Клетки опухоли не экспрессируют маркеры коры надпочечника (Melan A, Inhibin A), а также мозгового вещества (Chromogranin A). В опухолевой ткани отмечаются обширные участки некроза. Описанные гистологические особенности и иммунофенотип опухоли наиболее вероятно соответствуют метастазу лимфоэпителиомоподобной карциномы мочевого пузыря (lymphoepithelioma-like carcinoma, LELC) в надпочечник.

**Figure:**
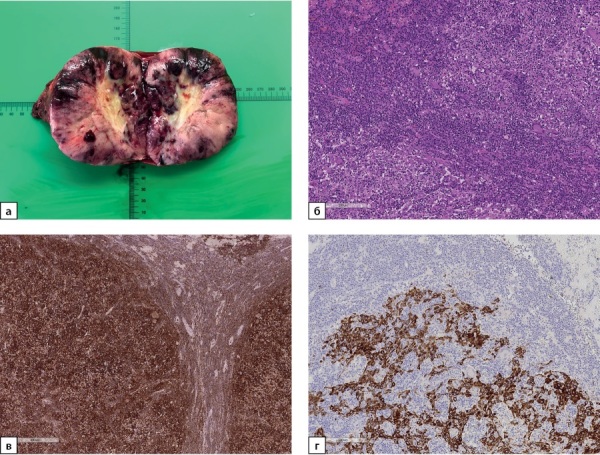
Рисунок 4а. Макропрепарат надпочечника с метастазом лимфоэпителиомоподобной карциномы мочевого пузыря диаметром 4,0 см.Рисунок 4б. Метастаз лимфоэпителиомоподобной карциномы в надпочечник. Опухоль представлена крупными эпителиальными клетками с бледной цитоплазмой, строма из нежноволокнистой соединительной ткани,инфильтрированной лимфоидными элементами. Ув. ×100. Окраска гематоксилином и эозином.Рисунок 4в. Метастаз лимфоэпителиомоподобной карциномы в надпочечник. Позитивное окрашивание клеток опухоли в реакции с MCK (общий цитокератин). Ув. ×100. ИГХ-реакция с антителом MCK.Рисунок 4г. Метастаз лимфоэпителиомоподобной карциномы в надпочечник. Позитивное окрашивание инфильтрирующих опухоль иммунных клеток в реакции с CD45 (общий лейкоцитарный антиген). Ув. ×100. ИГХ-реакция с антителом CD45.

## КЛИНИЧЕСКИЙ СЛУЧАЙ №5

Пациент В., 76 лет. В течение трех лет отмечал жалобы на потерю массы тела на 20 кг, боли в поясничной области, озноб в вечернее время, субфебрильную температуру в течение последних 5 мес, перемежающуюся хромоту в левой ноге. По данным УЗИ брюшной полости было выявлено объемное образование левого надпочечника. Проведена МСКТ органов брюшной полости, по результатам которой выявлено солидное образование левого надпочечника с ровными четкими контурами (предположительно нейрофиброма), нативной плотностью +23…+32 HU, размерами 60×56×53 мм, неоднородной структуры за счет единичных мелких кальцинатов. При динамическом наблюдении через 6 мес отмечена отрицательная динамика в виде увеличения размеров образования до 86×82×82 мм с нативной плотностью +33…+45 HU, накапливающего контраст по фазам сканирования артер.-венозн.-отсрочен.: 48–58–62 HU. Количественная лимфаденопатия парааортальных лимфатических узлов. По данным лабораторных исследований исключена гормональная активность образования надпочечника: 1) в суточной моче, собранной с консервантом, метанефрин — 186 мкг/сут (25–312), норметанефрин — 453 мкг/сут (35–445); 2) АКТГ базальный — 32 пг/мл (7–66), кортизол на фоне ночного подавляющего теста с 1 мг дексаметазона — 32 пг/мл (менее 50). Исключение первичного гиперальдостеронизма не требовалось в связи с отсутствием артериальной гипертензии. При поступлении, а также в ходе госпитализации у пациента отмечалось повышение температуры до 38,3°С. В ходе обследования исключены инфекционные и воспалительные причины лихорадки. По данным общеклинического анализа крови отмечался лейкоцитоз до 15,5×10⁹ кл./л, повышение СОЭ до 91 пг/мл.

Интраоперационно в брюшной полости у верхнего полюса почки выявлено опухолевидное образование плотной консистенции размерами 10 см в диаметре. В связи с высоким злокачественным потенциалом опухоли проведена лапароскопическая левосторонняя адреналэктомия.

Результаты патолого-анатомического исследования: в исследуемом постоперационном материале ткани надпочечника определяется разрастание опухоли преимущественно солидного строения, местами с образованием железистоподобных структур. Опухолевые клетки крупные, преимущественно с эозинофильной цитоплазмой и крупными полиморфными ядрами с крупноглыбчатым хроматином и одним или несколькими хорошо заметными ядрышками. Строма представлена тонкими, местами гиалинизированными фиброваскулярными септами, обращает на себя внимание выраженная лимфоцитарная инфильтрация стромы (рис. 5а).

Результаты ИГХ-исследования: в опухоли отмечаются выраженная диффузная реакция СК7 (рис. 5б), САМ 5.2, мезотелина (рис. 5в), очаговая экспрессия кальретинина и виментина (рис. 5г), также обращает на себя внимание очаговая позитивная реакция с ингибином. Выявлена экспрессия PDL1 в 1% опухолевых клеток. Индекс пролиферации Ki67 составил до 30%. Отрицательная ИГХ-реакция с антителами против Melan A позволяет исключить адренокортикальный рак (АКР), отрицательная реакция с RCC, CD10, PAX8 исключает наличие опухоли почки, отрицательная реакция с TTF1 и напсином исключает метастазы рака легкого. Описанная картина и иммунофенотип опухоли соответствуют локализованной злокачественной мезотелиоме.

Со 2-х суток после оперативного вмешательства отмечалась стойкая нормализация температуры тела.

**Figure:**
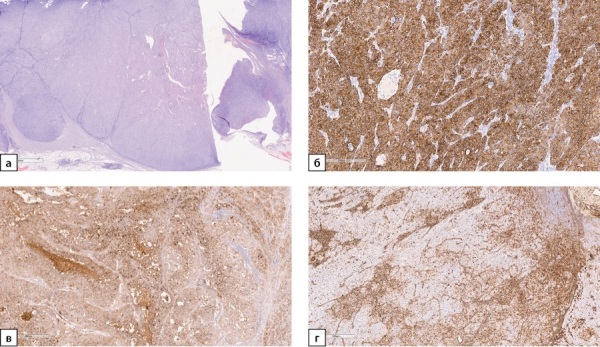
Рисунок 5а. Метастаз злокачественной мезотелиомы в надпочечник. Опухоль представлена крупными клетками. Фиброваскулярная строма с выраженной лимфоцитарной инфильтрацией. Ув. ×60. Окраска гематоксилином и эозином.Рисунок 5б. Метастаз злокачественной мезотелиомы в надпочечник. Позитивное окрашивание клеток опухоли в реакции с цитокератином 7 (CK7). Ув. ×200. ИГХ-реакция с антителом CK7.Рисунок 5в. Метастаз злокачественной мезотелиомы в надпочечник. Позитивное окрашивание клеток опухоли в реакции с мезотелином. Ув. ×100. ИГХ реакция с антителом к мезотелину.Рисунок 5г. Метастаз злокачественной мезотелиомы в надпочечник. Очаговая экспрессия виментина опухолевыми клетками. Ув. ×100. ИГХ реакция антителом к виментину.

## ОБСУЖДЕНИЕ

Согласно методическим рекомендациям, среди новообразований надпочечников выделяют: 1) адренокортикальные образования: рак или аденома; 2) опухоли мозгового вещества надпочечников: феохромоцитома; 3) метастатическое поражение надпочечников; 4) другие образования: кисты, липомы, тератомы и прочие; 5) «псевдонадпочечниковые» образования: опухоли почек, поджелудочной железы, селезенки, желудка, правой доли печени, забрюшинных лимфатических узлов или кровеносных сосудов [4].

Одним из ключевых факторов в выборе тактики лечения является установление наличия гормональной активности и определение злокачественного потенциала опухоли, поэтому всем пациентам с новообразованиями надпочечников рекомендовано проведение лабораторных и визуализирующих исследований.

Согласно данным литературы, метастазы в надпочечники обычно бессимптомны, однако встречаются случаи, которые проявляются болью в поясничной области, ретроперитонеальными кровотечениями или надпочечниковой недостаточностью [5]. В представленных нами случаях только у одного пациента с локализованной злокачественной мезотелиомой наблюдались клинические симптомы в виде потери массы тела, болей в поясничной области, озноба и температуры, в остальных случаях метастатическое поражение надпочечников не имело какой-либо клинической симптоматики.

Время между обнаружением первичной опухоли и возникновением метастазов в надпочечники зависит от локализации первичного очага и может значительно варьировать, в среднем составляя около 2,5 года [2]. Нередко изолированные метастазы в надпочечники возникают до выявления первичного злокачественного новообразования, как в случаях с метастазами светлоклеточного ПКР и мезотелиомы плевры, представленных в данном исследовании.

В исследовании J. Mao и соавт. (2020 г.) было проанализировано 579 случаев метастазирования в надпочечники. Чаще в надпочечниках обнаруживаются метастазы опухолей легких (226, 39,0%), мочеполовой системы (160, 27,6%), желудочно-кишечного тракта (79, 13,6%) и других (114, 19,7%) систем органов. Интересно, что 210 (36,3%) были обнаружены случайно, и лишь в 29 (5,0%) случаях обследование проводилось на основании клинических симптомов [5]. В нашем исследовании мы сосредоточились на случаях диагностики опухолей, которые, согласно данным литературы, метастазируют в надпочечники крайне редко, ввиду чего диагностика таких опухолей имеет множество трудностей.

В большинстве случаев биопсия надпочечника малоинформативна, а при АКР строго противопоказана. При проведении биопсии происходит разрыв капсулы узла, что в случае злокачественного новообразования приводит к диссеминации процесса и ухудшению прогноза заболевания, поэтому пункционная биопсия может быть применима только при неоперабельной опухоли в случае, если результат патоморфологического исследования может повлиять на тактику лечения пациентов [4][6]. Тем не менее в метаанализе 32 исследований I. Bancos и соавт. (2016 г.) приводят данные по обследованиям 2174 пациентов с инциденталомами надпочечников. Было проанализировано 2190 биопсий, средний диаметр опухолей составил 3,9 см. В 74% (n=1621) исследований была выявлена патология надпочечников: 689 (42,5%) случаев метастазов, 464 (28,6%) — аденом, 68 (4,2%) — АКР, 64 (3,9%) — других злокачественных опухолей, 226 (14%) — других доброкачественных опухолей, 36 (2,2%) — феохромоцитом и 74 (4,6%) — других опухолей [7].

На сегодняшний день адреналэктомия является основным методом лечения при подозрении на АКР или метастатическое поражение надпочечников. При проведении адреналэктомии предпочтительным доступом считается лапароскопический [8]. Согласно данным литературы, хирургическое вмешательство по поводу метастатических поражений надпочечников положительно влияет на прогноз пациентов (медиана выживаемости после адреналэктомии — 31,0 мес, без хирургического вмешательства — 8,5 мес) [9][10].

Зачастую диагноз метастатического поражения надпочечников удается установить лишь после патолого-анатомического исследования послеоперационного материала. Однако базовой окраски гематоксилином и эозином недостаточно для проведения дифференциальной диагностики, особенно при изучении опухолей надпочечников без установленного первичного очага. Наиболее тяжелой задачей в случае выявления опухоли надпочечников является дифференциальная диагностика с АКР ввиду высокой степени морфологической гетерогенности как в случаях различных опухолей, так и в пределах одного новообразования [11].

АКР — это редкая злокачественная эндокринная опухоль коры надпочечников c распространенностью 1–2 случая в год на 1 млн населения. Согласно классификации ВОЗ, выделяют несколько морфологических вариантов АКР. При этом даже в пределах классического гистологического варианта идентифицированы различные морфологические особенности: преобладание крупных полиморфных клеток, мономорфных клеток малого или среднего размера («карциноидоподобных») или рабдоидных клеток, формирующих альвеолярные структуры [12].

SF-1 — ядерный транскрипционный фактор, регулирующий выработку стероидных гормонов в коре надпочечников, является наиболее чувствительным (98%) и специфичным (100%) маркером АКР. При подозрении на АКР в обязательном порядке необходимо проведение серии иммуногистохимических исследований. В основную панель маркеров, позволяющих исключить или подтвердить диагноз АКР, на сегодняшний день также принято включать Melan A и Inhibin A, однако их использование не столь специфично [13].

Отсутствие экспрессии SF-1 позволяет исключить корковый гистогенез опухоли надпочечников. ИГХ-реакция с SF-1 обычно отрицательна при ПКР, гепатоцеллюлярной карциноме, меланоме и феохромоцитоме. Для проведения дифференциальной диагностики с феохромоцитомой целесообразно использование Chromogranin A, который экспрессируется исключительно клетками мозгового слоя надпочечников [14].

В аутопсийном исследовании более 400 пациентов, перенесших нефрэктомию по поводу ПКР, контралатеральный надпочечник был единственным местом метастазирования только у 2,5%. Среди пациентов с распространенными метастазами ПКР, выявленными при аутопсии, контралатеральный надпочечник был поражен у 12,7% пациентов [15].

При подозрении на метастазы ПКР в ИГХ-панель рационально включать такие маркеры, как PAX2, PAX8, RCC, panCK, виментин, CK7. PAX2 имеет широкий диапазон экспрессии и обнаруживается при большинстве почечно-клеточных карцином, за исключением хромофобного варианта. CD10 положителен в большинстве светлоклеточных и папиллярных почечно-клеточных карцином [16].

Метастазы ПРЩЖ чаще возникают в регионарных лимфатических узлах. Отдаленные метастазы встречаются редко и обычно затрагивают легкие или кости. Для ткани щитовидной железы и карцином щитовидной железы характерна экспрессия TTF-1 и тиреоглобулина. Фолликулярный, папиллярный и медуллярный рак щитовидной железы, как правило, сильно положительны на TTF-1, тогда как недифференцированный (анапластический) — чаще всего отрицательный [17]. Напсин А экспрессируется в большинстве аденокарцином легких и используется в качестве специфического маркера.

Описания случаев метастатического поражения надпочечников вследствие гинекологических новообразований, найденные в литературе, немногочисленны: опубликовано когортное исследование с участием 34 пациентов, которое описывает 2 случая (6%) метастазирования карциномы шейки матки в надпочечники [18]. Для подтверждения предположения о метастазе ороговевающей плоскоклеточной карциномы шейки матки нами были использованы p63 и CK5/6, которые являются традиционными маркерами, указывающими на плоскоклеточную дифференцировку [19].

Лимфоэпителиомоподобная карцинома мочевого пузыря — редкий гистологический тип злокачественной опухоли, составляющий 0,4–1,3% всех случаев рака мочевого пузыря [20]. Гистологические особенности данной опухоли включают воспалительный инфильтрат и плотный лимфоцитарный инфильтрат, кроме того, при гистологическом исследовании данной опухоли можно наблюдать синцитиальное расположение крупных неопластических эпителиальных клеток с выступающими ядрами и ядрышками. Несмотря на инфильтративную предрасположенность, метастатический потенциал данной опухоли представляется низким [21]. Для установления окончательного диагноза необходимо ИГХ-выявление цитокератинов, которые доказывают эпителиальное происхождение опухоли. В представленном случае для выявления метастаза лимфоэпителиомоподобной карциномы мочевого пузыря в надпочечник были использованы маркеры MCK (общий цитокератин) и CD45 (общий лейкоцитарный антиген) [22].

Серия патологоанатомических исследований 318 пациентов показала, что чаще метастазы мезотелиомы плевры выявляются в печени (32%), селезенке (11%), щитовидной железе (7%) и головном мозге (3%) [23]. При этом в литературе зарегистрировано всего несколько случаев распространения мезотелиомы в надпочечники, для которой характерно наличие ряда маркеров: кальретинин, антиген WT-1, виментин, мезотелин [24].

## ЗАКЛЮЧЕНИЕ

Дифференциальная диагностика между доброкачественными, первичными и вторичными злокачественными опухолями является критической проблемой как для врачей клинических специальностей, так и для морфологов, особенно у пациентов с изолированными поражениями надпочечников.

Во всех представленных нами случаях в ходе диагностического поиска на дооперационном этапе результаты лабораторных и инструментальных обследований так или иначе указывали на возможный диагноз АКР. Однако установить окончательный диагноз удалось только на основании ИГХ-исследований.

Как известно, решающее влияние на выживаемость оказывает терапия. Наличие метастазов влияет на тактику лечения первичного злокачественного новообразования, и часто требуется дальнейшее обследование, особенно у пациентов с раком, у которого нет других локализаций метастазов, кроме надпочечников. Таким образом, неправильный диагноз может привести к неправильному лечению опухолей надпочечников: чрезмерному лечению или игнорированию.

Из-за морфологической гетерогенности АКР бывают случаи, когда практически невозможно отличить метастазы от первичного рака надпочечников при стандартной окраске гематоксилином и эозином. Именно поэтому при исследовании опухолей надпочечников необходимо соблюдать настороженность и в обязательном порядке использовать весь спектр современных методов, в частности ИГХ-исследования, чтобы избежать ошибочных диагнозов.

## ДОПОЛНИТЕЛЬНАЯ ИНФОРМАЦИЯ

Источники финансирования. Работа выполнена по инициативе авторов без привлечения финансирования.

Конфликт интересов. Авторы декларируют отсутствие явных и потенциальных конфликтов интересов, связанных с содержанием настоящей статьи.

Участие авторов. Все авторы внесли значимый вклад в подготовку статьи, прочли и одобрили финальную версию статьи перед публикацией, выразили согласие нести ответственность за все аспекты работы, подразумевающую надлежащее изучение и решение вопросов, связанных с точностью или добросовестностью любой части работы.

Согласие пациента. Пациентами была подписана форма информированного согласия на публикацию персональной медицинской информации в обезличенной форме в журнале «Проблемы эндокринологии».
